# A Novel Photo-Driven Hydrogenation Reaction of an NAD^+^-Type Complex Toward Artificial Photosynthesis

**DOI:** 10.3389/fchem.2019.00580

**Published:** 2019-08-20

**Authors:** Hideki Ohtsu, Tsubasa Saito, Kiyoshi Tsuge

**Affiliations:** Graduate School of Science and Engineering, University of Toyama, Toyama, Japan

**Keywords:** CO_2_ reduction, photo-induced hydrogenation, NAD^+^, NADH, organic hydride, ruthenium complex

## Abstract

The photocatalytic reduction of carbon dioxide (CO_2_) to value-added chemicals is an attractive strategy to utilize CO_2_ as a feedstock for storing renewable energy, such as solar energy, in chemical bonds. Inspired by the biological function of the nicotinamide adenine dinucleotide redox couple (NAD^+^/NADH), we have been developing transition-metal complexes containing NAD^+^/NADH-functionalized ligands to create electro- and/or photochemically renewable hydride donors for the conversion of CO_2_ into value-added chemicals. Our previous findings have provided insights for the development of photocatalytic organic hydride reduction reactions for CO_2_, however, further examples, as well as investigation, of these photo-driven NAD^+^/NADH-type hydrogenation and organic hydride transfer reactions are required not only to explore the mechanism in detail but also to develop a highly efficient catalyst for artificial photosynthesis. In this paper, we report the synthesis, characterization, and photo-induced NAD^+^/NADH conversion properties of a new ruthenium(II) complex, [Ru(bpy)_2_(Me-pn)](PF_6_)_2_ (**1**), which contains a new NAD^+^-type ligand, Me-pn (2-methyl-6-(pyridin-2-yl)-1,5-naphthyridine). In addition, we have succeeded in the isolation of the corresponding two-electron reduced ruthenium(II) complex containing the NADH-type ligand Me-pnHH (2-methyl-6-(pyridin-2-yl)-1,4-dihydro-1,5-naphthyridine), i.e., [Ru(bpy)_2_(Me-pnHH)](PF_6_)_2_ (**1HH**), by the photo-induced hydrogenation reaction of **1**. Thus, in this study, a new photo-driven NAD^+^/NADH-type hydrogenation reaction for possible CO_2_ reduction using the NAD^+^/NADH redox function has been constructed.

## Introduction

Currently, the design and development of novel visible-light photoredox catalysts for carbon dioxide (CO_2_) reduction are considered to be crucial challenges. In particular, it is vital not only to clarify how to employ solar energy, which is a sustainable clean energy source, but also to develop methods to convert CO_2_, which is a major greenhouse gas and is harmful for the environment, into chemical fuels and feedstocks in the viewpoint of the current global energy and environmental crisis (Fox and Dulay, 1993; Kisch, 2013). Many research efforts have so far been devoted to the search for new and efficient catalysts for CO_2_ conversion into value-added chemicals such as carbon monoxide (CO), formic acid (HCO_2_H), and methanol (CH_3_OH) (Meyer, 1989; Alstrum-Acevedo et al., 2005; Wang et al., 2015). However, there are several problems facing photochemical CO_2_ conversion, particularly related to the side reactions and low selectivity toward specific reduction products, as well as poor energy efficiency (Leitner, 1995; Jones et al., 2014). To solve these problems, the use of transition-metal coordination compounds as photocatalysts for CO_2_ reduction has drawn significant attention because some of these compounds exhibit significant photocatalytic activity for CO_2_ reduction (Morris et al., 2009; Berardi et al., 2014), and they play an essential role in artificial photosynthesis (Fukuzumi et al., 2018), as well as natural photosynthesis (Silva et al., 2015).

In particular, ruthenium complexes are promising candidates for photocatalysts because most Ru complexes show excellent photophysical properties with relatively long excited-state lifetimes and visible light absorption bands originating from metal-to-ligand charge transfer (MLCT) (Medlycott and Hanan, 2005; Sun et al., 2015; Dongare et al., 2017). In the past several decades, a number of Ru complexes having photocatalytic ability toward CO_2_ reduction have been reported (Jessop et al., 1995; Tanaka and Ooyama, 2002; Kuramochi et al., 2018). In this context, we have focused on Ru complexes having NAD^+^/NADH-functionalized ligands because the biological function of the NAD^+^/NADH redox couple is as a generator and reservoir of hydride ions (H^−^), which are equivalent to two electrons and one proton (Eisner and Kuthan, 1972; Walsh, 1980; Stout and Meyers, 1982; Gebicki et al., 2004; Bilan et al., 2015), and is of great interest for the development of photorenewable hydride reagents.

Previously, we have successfully demonstrated that a ruthenium(II) complex containing an NADH-type ligand, pbnHH [Ru(bpy)_2_(pbnHH)]^2+^ (bpy = 2,2′-bipyridine, pbnHH = 2-(pyridin-2-yl)-5,10-dihydrobenzo[*b*][1,5]naphthyridine), which is photochemically converted from the corresponding NAD^+^-type complex [Ru(bpy)_2_(pbn)]^2+^ (pbn = 2-(pyridin-2-yl)benzo[*b*][1,5]naphthyridine) (Koizumi and Tanaka, 2005; Polyansky et al., 2007; Fukushima et al., 2009, 2010; Ohtsu and Tanaka, 2012a), can reduce CO_2_ to ${\text{HCO}}_{2}^{-}$ via organic hydride transfer involving C–H bond dissociation of the NADH-type ligand in [Ru(bpy)_2_(pbnHH)]^2+^ driven by the association of the bases (Ohtsu and Tanaka, 2012b; Ohtsu et al., 2015). These findings stimulated us to explore a new type of NAD^+^/NADH-functionalized complex, and the expansion of the scope of our previous work on NAD^+^/NADH model chemistry potentially opens new perspectives for the design of more efficient molecular photocatalysts for CO_2_ reduction. As part of our ongoing research into Ru NAD^+^/NADH-functionalized complexes, we have synthesized and characterized the photo-induced NAD^+^/NADH-type hydrogenation reaction properties of a ruthenium(II) complex bearing a new NAD^+^-type ligand, Me-pn (Me-pn = 2-methyl-6-(pyridin-2-yl)-1,5-naphthyridine), i.e., [Ru(bpy)_2_(Me-pn)](PF_6_)_2_ (**1**). In addition, we have also successfully isolated a ruthenium(II) complex having the corresponding NADH-type ligand, Me-pnHH (Me-pnHH = 2-methyl-6-(pyridin-2-yl)-1,4-dihydro-1,5-naphthyridine), i.e., [Ru(bpy)_2_(Me-pnHH)](PF_6_)_2_ (**1HH**), by the photochemical reduction of **1** under light irradiation (λ > 420 nm) in the presence of a sacrificial reagent.

## Materials and Methods

### Materials

All chemicals used for the synthesis of the ligands and complexes were commercial products of the highest available purity and were used without further purification unless otherwise indicated. Solvents were purified by standard methods before use (Armarego and Chai, 2009).

### Synthesis

All ligands and complexes used in this study were prepared according to the following procedures and the structures of the products were confirmed from analytical data (*vide infra*).

### 2-Methyl-6-(pyridin-2-yl)-1,5-naphthyridine (Me-pn)

This ligand was prepared in the same manner as that for the synthesis of 2-(pyridin-2-yl)-1,5-naphthyridine (Singh and Thummel, 2009) using 2-chloro-6-methyl-1,5-naphthyridine (Gellibert et al., 2004) instead of 2-chloro-1,5-naphthyridine. Anal. Calcd for C_14_H_11_N_3_: C, 76.00; H, 5.01; N, 18.99. Found: C, 75.84; H, 4.88; N, 18.81. ^1^H NMR (300 MHz, CDCl_3_): δ 8.73 ~ 8.79 (m, 2H), 8.60 (dt, *J* = 8.0, 1.1 Hz, 1H), 8.44 (d, *J* = 8.8 Hz, 1H), 8.35 (d, *J* = 8.6 Hz, 1H), 7.88 (td, *J* = 7.8, 2.0 Hz, 1H), 7.54 (d, *J* = 8.6 Hz, 1H), 7.38 (ddd, *J* = 7.4, 4.8, 1.3 Hz, 1H), 2.81 (s, 3H).

### [Ru(bpy)_2_(Me-pn)](PF_6_)_2_ (1)

To a 2-methoxyethanol solution (20 mL) of [Ru(bpy)_2_Cl_2_] (363 mg, 0.75 mmol) was added AgPF_6_ (381 mg, 1.5 mmol) in 2-methoxyethanol, and the mixture was stirred for 12 h at 70°C. The resulting mixture was cooled to room temperature, and insoluble material was removed by filtration through celite. After the addition of the ligand Me-pn (754 mg, 0.75 mmol) to the filtrate, the reaction mixture was stirred for 24 h at 70°C. The solution was concentrated to ca. 1 mL and poured into aqueous NH_4_PF_6_ solution. The solid formed was collected and dried in vacuo. Recrystallization from acetonitrile/diethylether gave **1** as reddish purple crystals (389 mg, 56.1%). ESI-MS: *m/z* = 780 [*M*–PF_6_]^+^. Anal. Calcd for C_34_F_12_H_29_N_7_OP_2_Ru: C, 43.32; H, 3.10; N, 10.40. Found: C, 43.48; H, 3.25; N, 10.30. ^1^H NMR(300 MHz, CD_3_CN): δ 8.73 (d, *J* = 9.1 Hz, 1H), 8.72 (d, *J* = 8.2 Hz, 1H), 8.51 ~ 8.61 (m, 3H), 8.38 (d, *J* = 8.2 Hz, 1H), 8.30 (d, *J* = 8.1 Hz, 1H), 8.18 ~ 8.32 (m, 5H), 7.89 ~ 7.99 (m, 2H),7.67 (d, *J* = 5.6 Hz, 1H), 7.58 (d, *J* = 5.7 Hz, 1H), 7.38 ~ 7.53 (m, 4H), 7.27 ~ 7.38 (m, 3H), 7.11 (d, *J* = 9.1 Hz, 1H), 2.62 (s, 3H).

### [Ru(bpy)_2_(Me-pnHH)](PF_6_)_2_ (1HH)

The NADH-type two-electron reduced complex was prepared photochemically: An anaerobic CH_3_CN solution (2.35 mL) of **1** (30.7 mg, 0.033 mmol) containing TEOA (0.208 g, 1.4 mmol) was irradiated with light through a longpass filter (HOYA W-Y455) and a super cold filter (ASAHI SPECTRA SC0751) using a 150 W Xenon lamp for 0.5 h. The resulting solution was added to aqueous NH_4_PF_6_ solution, and the orange solid formed was collected by filtration and dried in vacuo. Yield: 24.2 mg (78.5%). ESI-MS: *m/z* = 782 [*M*–PF_6_]^+^. Anal. Calcd for C_34_F_12_H_29_N_7_P_2_Ru: C, 44.07; H, 3.15; N, 10.58. Found: C, 43.95; H, 3.21; N, 10.36. ^1^H NMR(300 MHz, CD_3_CN): δ 8.52 (d, *J* = 8.3 Hz, 2H), 8.44 (t, *J* = 7.1 Hz, 2H), 8.18 ~ 8.26 (m, 2H), 8.06 ~ 8.15 (m, 3H), 7.89 ~ 8.05 (m, 3H), 7.64 ~ 7.72 (m, 2H), 7.52 (td, *J* = 6.4, 1.4 Hz, 1H), 7.42 ~ 7.48 (m, 2H), 7.24 ~ 7.37 (m, 3H), 7.16 (td, *J* = 6.6, 1.3 Hz, 1H), 7.10 (d, *J* = 8.7 Hz, 1H), 6.68 (br, s, 1H), 3.85 (br, m, 1H), 3.36 (ddd, *J* = 21.5, 3.9, 1.6, Hz, 1H), 2.35 (ddd, *J* = 21.5, 3.4, 1.8 Hz, 1H), 1.62 (br, m, 3H).

### Physical Measurements

All manipulations were carried out under an argon atmosphere using standard Schlenk techniques or in a glovebox. The absorption spectra were obtained using a Hewlett–Packard 8453 diode array spectrophotometer or a Shimadzu UV-3100PC scanning spectrophotometer. The emission spectra were recorded on a JASCO FP-8500 spectrofluorometer. The emission lifetimes were performed with a UNISOKU LSP-1000N-PX spectrometer. The electrospray (ESI)-mass spectrometry (MS) data were obtained with a Shimadzu LCMS-2020. Elemental analyses were performed using a Yanaco CHN Corder MT-5 (A Rabbit Science Japan Co., Ltd.). NMR measurements were measured with a JEOL JMN-ECX 300 (300 MHz) NMR spectrometer. Electron spin resonance (ESR) spectra were taken with a JEOL JES-X310 equipped with a variable temperature (VT) apparatus and were recorded under non-saturating microwave power conditions. The magnitude of the modulation was chosen to optimize the resolution and the signal-to-noise ratio of the observed spectra. The *g* values were calibrated with a Mn(II) marker used as a reference. Cyclic voltammetry measurements were performed on an ALS/Chi model 733D electrochemical analyzer in a deaerated solvent containing 0.1 M tetra-*n*-butylammonium hexafluorophosphate (TBAPF_6_) as a supporting electrolyte. A conventional three-electrode cell was used with a glassy-carbon working electrode and a platinum wire as the counter electrode. The glassy-carbon working electrode was routinely polished with a BAS polishing alumina suspension and rinsed with acetone before use. The reversibility of the electrochemical processes was evaluated by standard procedures, and all potentials were recorded against an Ag/AgCl reference electrode. All electrochemical measurements were carried out under an argon atmosphere.

### X-ray Crystal Structure Determination

The single crystal X-ray diffraction data of **1**•CH_3_CN•Et_2_O were collected on a Rigaku VariMax RAPID-DW/NAT with Mo-*K*α radiation (λ = 0.71075 Å) at 173 K and processed using RapidAuto program (Rigaku). An empirical absorption correction resulted in acceptable transmission factors. The data were corrected for Lorentz and polarization factors. The structure was solved by direct methods using SHELXT-2018/2 (Sheldrick, 2015) and refined by full-matrix least-squares techniques on *F*^2^ using SHELXL-2018/3 (Sheldrick, 2015). All non-hydrogen atoms were refined anisotropically and all hydrogen atoms were included in the refinement process as per the riding model. CCDC-1894384 contains the supplementary crystallographic data for this paper. These data can be obtained free of charge via www.ccdc.cam.ac.uk/conts/retrieving.html (or from the Cambridge Crystallographic Data Center, 12, Union Road, Cambridge CB21EZ, UK; fax: (+44)1223-336-033; or deposit@ccdc.cam.ac.uk).

## Results and Discussion

The ruthenium(II) complex, [Ru(bpy)_2_(Me-pn)](PF_6_)_2_ (**1**), which bears our newly designed NAD^+^-type ligand, 2-methyl-6-(pyridin-2-yl)-1,5-naphthyridine (Me-pn), was successfully prepared by mixing [Ru(bpy)_2_Cl_2_] and AgPF_6_ in a 1:2 ratio in 2-methoxyethanol, followed by the addition of 1 equivalent of Me-pn at 343 K, and the composition of **1** was determined by elemental analysis, ESI-MS, and ^1^H-NMR (see Material and Methods).

**Figure 1:**
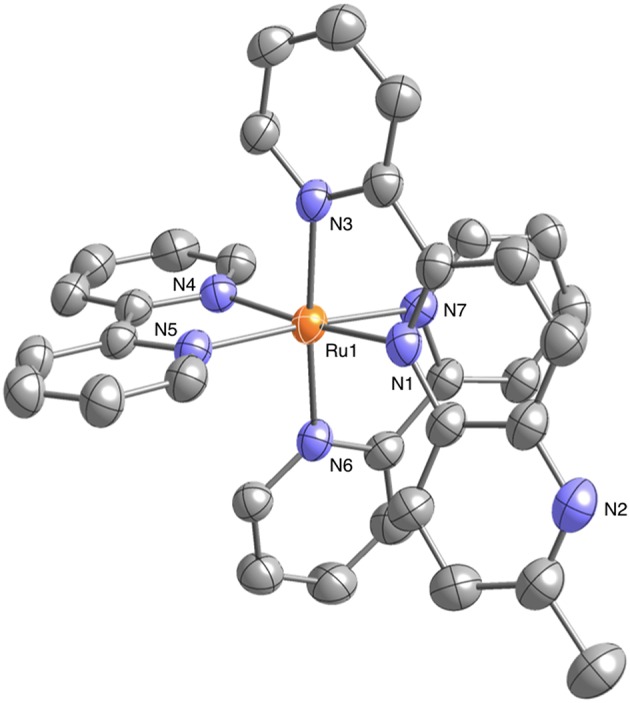
Crystal structure of the ruthenium(II) NAD^+^-type complex [Ru(bpy)_2_(Me-pn)](PF_6_)_2_•CH_3_CN•Et_2_O (**1**•CH_3_CN•Et_2_O) (50% probability ellipsoids. orange: ruthenium, blue: nitrogen, gray: carbon). The counter anion (${\text{PF}}_{6}^{-}$), hydrogen atoms, and CH_3_CN and Et_2_O molecules are omitted for clarity.

Well-shaped single crystals of **1** suitable for X-ray structure determination were obtained by recrystallization from an acetonitrile (CH_3_CN) solution using the slow vapor diffusion of diethylether (Et_2_O). This compound crystallizes in the monoclinic *P*2_1_/*n* space group with four molecules in the unit cell. The structure also contains solvated CH_3_CN and Et_2_O for each complex. The molecular structure of **1** is shown in Figure 1. The ruthenium(II) ion in **1** has a hexacoordinate octahedral structure formed of four N atoms of the two bpy ligands and two N atoms of the Me-pn ligand. The coordination environment of **1** is almost the same as that of a previously reported ruthenium(II) NAD^+^-type complex [Ru(bpy)_2_(pbn)](PF_6_)_2_ (Koizumi and Tanaka, 2005), and the bond lengths between the ruthenium(II) ion and the six N atoms of the ligands for **1** (2.046(4) to 2.119(4) Å) are not only the same as those of [Ru(bpy)_2_(pbn)](PF_6_)_2_ (2.038(5) to 2.116(5) Å) (Koizumi and Tanaka, 2005) but also within the typical range of those of reported ruthenium(II) complexes having a similar coordination environment (Fukushima et al., 2009, 2010; Ohtsu and Tanaka, 2012a; Kobayashi et al., 2016). The ESI-MS results for **1** in CH_3_CN show a dominant signal at *m/z* = 780, as shown in Figure S1, and the observed mass value, as well as the isotopic pattern, agree well with those of the simulated pattern of [Ru(bpy)_2_(pbn)](PF_6_)]^+^. In addition, the ^1^H-NMR spectrum of **1** in CD_3_CN (Figure S2) displays a spectral pattern reflecting the *Cs* symmetry of **1**, including 24 aromatic proton signals between 7.11 and 8.73 ppm and three proton singlet signal assigned to the methyl group at 2.62 ppm. These results are in complete agreement with the single-crystal X-ray diffraction analysis described above.

**Figure 2:**
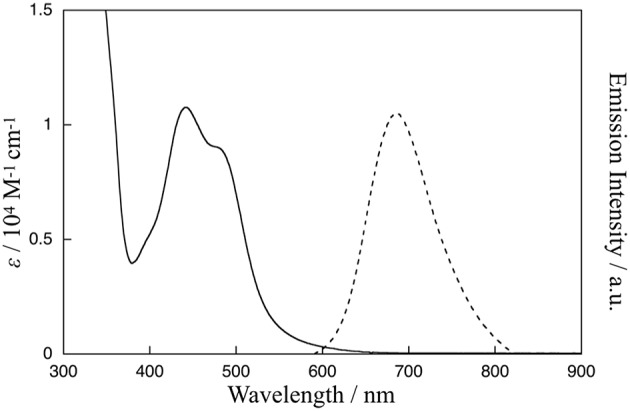
Absorption spectrum (solid line) of **1** in CH_3_CN at 298 K and emission spectrum (dotted line) of **1** in CH_3_CN at 298 K upon excitation at 480 nm.

The absorption and emission spectra of **1** in CH_3_CN at 298 K are shown in Figure 2. Characteristic absorption bands centered around 440 and 480 nm can be assigned to MLCT transitions from the ruthenium(II) centers to the bpy ligand (Caspar and Meyer, 1983) and the Me-pn ligand, respectively. Complex **1** also exhibits a ^3^MLCT emission band around 685 nm upon excitation at 480 nm, as shown in Figure 2. Additionally, the emission lifetime has been evaluated from the resulting single exponential emission decay (368 ns, see Figure S3), and this emission lifetime is larger than that of a previously reported ruthenium(II) NAD^+^-type complex [Ru(bpy)_2_(pbn)](PF_6_)_2_ (140 ns) (Polyansky et al., 2007). Furthermore, the emission quantum yield (λ_ex_ = 480 nm) was estimated at ø = 0.37% using a relative method (Eaton, 1988) with [Ru(bpy)_3_](PF_6_)_2_ as a reference (0.095) (Suzuki et al., 2009; Ishida et al., 2010). This value is more than 500 times larger than that of [Ru(bpy)_2_(pbn)](PF_6_)_2_ (0.00071%) (Ohtsu and Tanaka, 2012a) reported previously.

**Figure 3:**
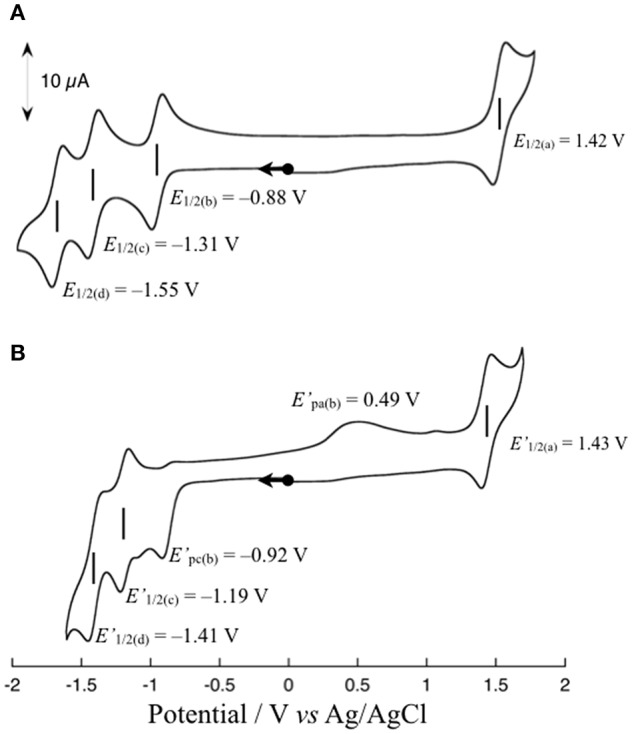
Cyclic voltammograms of **1** in the absence **(A)** and presence **(B)** of 10 equivalents of AcOH in DMF containing 0.1 M TBAPF_6_ at a scan rate 50 mV s^−1^. All scans are started from the rest potential.

Figure 3A shows the cyclic voltammogram (CV) of **1** (1 mM) in dimethylformamide (DMF) at 298 K. The CV shows one reversible redox wave at *E*_1/2(a)_ = 1.42 V (vs. Ag/AgCl) in the range above the rest potential (*E*_rest_ = 0.00 V) and three reversible redox waves at *E*_1/2(b)_ = −0.88 V, *E*_1/2(c)_ = −1.31 V, and *E*_1/2(d)_ = −1.55 V in the range below *E*_rest_ = 0.00 V. The *E*_1/2(a)_ redox wave can be assigned to the Ru(II)/Ru(III) couple on the basis of the rest potential. To clarify the assignment of the other three redox waves, the CV of **1** upon the addition of an excess amount of a proton source such as acetic acid (AcOH) was measured in accordance with the previously reported procedure (Kobayashi et al., 2016). The CV changes observed upon the addition of 10 equivalents of AcOH into a solution of **1** in DMF are shown in Figure 3B. As shown, the reversibility of the *E*_1/2(b)_ redox wave changes drastically to an irreversible cathodic peak at $E\prime_{\text{pc}(\text{b})}$ = −0.92 V, and a new anodic peak at $E\prime_{\text{pa}(\text{b})}$ = 0.49 V coupled with an irreversible $E\prime_{\text{pc}(\text{b})}$ peak appears, whereas the other two *E*_1/2(c)_ and *E*_1/2(d)_ redox waves are barely changed from the redox waves at $E\prime_{1/2(c)}$ = −1.19 V and $E\prime_{1/2(d)}$ = −1.41 V, respectively, maintaining redox reversibility. The lack of reversibility of the *E*_1/2(b)_ redox wave in the presence of the proton source is most likely caused by the protonation of the non-bonded N atom of the reduced Me-pn ligand; thus, the redox wave at *E*_1/2(b)_ = −0.88 V can be assigned to the Me-pn/Me-pn^•−^ couple. The large separation between the cathodic and anodic peak potentials of the Me-pn localized redox reaction under protic conditions may result from significant structural changes, such as the hydrogenation of the NAD^+^-type Me-pn ligand to form the NADH-type Me-pnHH ligand through a two electron and two proton process. In the case of the other two redox waves (*E*_1/2(c)_ = −1.31 V and *E*_1/2(d)_ = −1.55 V), it is safe to conclude that the *E*_1/2(c)_ and *E*_1/2(d)_ redox waves correspond to the redox couples of the two bpy ligands since almost no changes in the reversibility and the position of these redox waves are observed upon the addition of 10 equivalents of AcOH on the basis of CV measurements described above. In addition, the p*K*_a_ of the Me-pn ligand in **1** has been determined by spectrophotometric acid–base titration from pH 0.29 to pH 4.65, as shown in Figure S4. The estimated p*K*_a_ value of the protonated species [Ru(bpy)_2_(Me-pnH)]^3+^ is 1.6, the value of which is almost the same as previously reported in the case of [Ru(bpy)_2_(pbn)](PF_6_)_2_ (p*K*_a_ = 1.7) (Polyansky et al., 2007).

To estimate the potential photochemical reactivity of **1**, the excited state reduction potential must be known. The excited state reduction potential of **1** (*E*°([Ru(bpy)_2_(Me-pn)]^2^+^^*^/+^)) is correlated with *E*_1/2_(Me-pn/Me-pn^•−^) and *E*_em_(0–0) and is calculated to be 1.00 V using equation (1), where *E*_1/2_(Me-pn/Me-pn^•−^) = −0.88 V and *E*_em_(0–0) = 1.88 eV, which was estimated from the ^3^MLCT emission band (λ_ex_ = 658 nm at 77 K in *n*-butyronitrile). The fact that the excited state reduction potential (*E*°([Ru(bpy)_2_(Me-pn)]^2^+^^*^/+^) = 1.00 V) is located at a more positive potential than the oxidation potential of triethanolamine (TEOA, *E*_ox_ = 0.80 V vs. SCE) (Kalyanasundaram, 1986) stimulated us to research the photochemical reactivity of **1**, such as the photo-driven NAD^+^/NADH-type hydrogenation reaction reported previously (Polyansky et al., 2007) in the presence of TEOA as a sacrificial reagent.

(1) $$\begin{array}{l} E^{{}^{\circ}}({\left[\text{Ru}{\left(\text{bpy}\right)}_{\text{2}}(\text{Me-pn})\right]}^{{\text{2+}}^{*}\text{/+}})\\ \;=E_{\text{1/2}}({\text{Me-pn/Me-pn}}^{•\text{-}})+E_{\text{em}}(0-0) \end{array}$$

**Figure 4:**
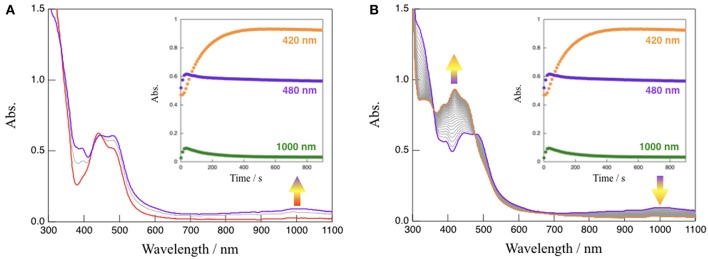
Absorption spectral changes observed upon the irradiation with visible light (λ > 420 nm) to a solution (CH_3_CN:TEOA = 4:1 v/v) of **1** at 293 K from 0 to 20 s **(A)** and from 20 to 900 s **(B)**, respectively. Inset: time course of the absorption changes at 420, 480, and 1,000 nm.

In the absence of TEOA, complex **1** in CH_3_CN was quite stable even when exposed to visible light (150-W Xe lamp, λ > 420 nm) (see Figure S5). However, absorption spectral changes were observed upon the irradiation with visible light to a solution of **1** in the presence of TEOA (CH_3_CN:TEOA = 4:1 v/v) at 293 K as shown in Figure 4. This reaction consists of two distinct steps. First, the characteristic absorption band located at 480 nm arising from **1** (red line) rapidly changes, and a spectrum (purple line) exhibiting a new broad band around 1,000 nm with an accompanying isosbestic point at 448 nm from 0 to 20 s is obtained; in addition, the intensity of the spectrum around 1,000 nm subsequently decreased. This is accompanied by an increase in the absorption band located at 420 nm (orange line) with an isosbestic point at 477 nm from 20 to 900 s. When triethylamine (TEA, *E*_ox_ = 0.66 V vs. SCE) (Yanagida et al., 1997), which is also a sacrificial reagent, was used instead of TEOA, almost the same absorption spectral changes indicating a two-step process have been observed (Figure S6), and a dominant signal (*m/z* = 782), which agrees well with the simulated pattern corresponding to the NADH-type complex, [Ru(bpy)_2_(Me-pnHH)](PF_6_)]^+^, has been detected by ESI-MS measurements of the final reaction mixture, as shown in Figure S7. Furthermore, attempts to isolate the NADH-type complex [Ru(bpy)_2_(Me-pnHH)](PF_6_)_2_ (**1HH**) by photochemical reduction using TEOA were successful based on the ESI-MS at *m/z* = 782 and the observed mass value, as well as the isotopic pattern, which agrees well with the simulated pattern of [Ru(bpy)_2_(Me-pnHH)](PF_6_)]^+^. In addition, 22 characteristic proton signals in the aromatic region, a proton N–H signal at 6.68 ppm, and two proton signals at 3.36 and 2.35 ppm, which can be assigned to the methylene protons of the 1,4-dihydropyridine frameworks in the Me-pnHH ligand (see Material and Methods and Figure S8), were observed. As reported for the previous ruthenium(II) NAD^+^-type complex [Ru(bpy)_2_(pbn)]^2+^, [Ru(bpy)_2_(pbn)]^2+^ is efficiently reduced to the corresponding NADH-type complex ([Ru(bpy)_2_(pbnHH)]^2+^) by photochemical reduction in CH_3_CN in the presence of a sacrificial electron donor such as TEOA or TEA. The absorption spectral changes for the photo-driven NAD^+^/NADH-type hydrogenation reaction in [Ru(bpy)_2_(pbn)]^2+^ system were observed in a one-step process (Ohtsu and Tanaka, 2012a) despite the use of the same experimental conditions as those of **1** (150-W Xe lamp(λ > 420 nm), CH_3_CN:TEOA = 4:1 v/v, 293 K). Such a difference in the reactivity may be ascribed to the steric effects of the methyl group of the Me-pn ligand in **1**, which result in weaker π-π interactions between two [Ru(bpy)_2_(Me-pnH^•^)]^2+^ (**1**^**•−**^**H**) radical intermediate species compared to the case of the non-substituted pbn ligand in [Ru(bpy)_2_(pbn)]^2+^; this is based on a possible mechanism for the photo-driven NAD^+^/NADH-type hydrogenation reaction discussed later.

**Figure 5:**
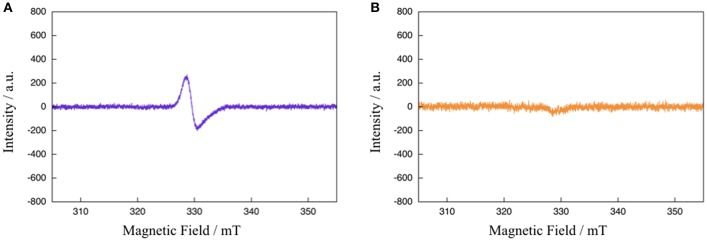
ESR spectra of **1** in CH_3_CN:TEOA = 4:1 v/v solution after photoirradiation (λ > 420 nm) for 20 s **(A)** and 900 s **(B)** at 173 K.

To gain further mechanistic insight into the photo-induced NAD^+^/NADH-type hydrogenation reaction of **1**, ESR measurements after photoirradiation for 20 and 900 s under the same conditions as those used to observe the absorption spectral changes shown in Figure 4 were carried out (Figure 5). The ESR spectrum of **1** in CH_3_CN:TEOA = 4:1 v/v solution after photoirradiation for 20 s at 173 K displays a pseudo-isotropic signal at *g* = 2.000 with a line width of Δ*H* = 19 G, as shown in Figure 5A. In contrast to the case of photoirradiation for 900 s, no ESR signal is observed (Figure 5B). The observed ESR signal in the case of photoirradiation for 20 s (Figure 5A) is similar to that of a one electron reduced [Ru(bpy)_3_]^2+^ complex, namely [Ru(bpy)_2_(bpy^•−^)]^+^ (*g* = 1.996 and Δ*H* = 26 G at 173 K) (Motten et al., 1981), which can be assigned to a ligand-centered radical species [Ru(bpy)_2_(Me-pnH^•^)]^2+^ (**1**^**•−**^**H**) based on a previous mechanistic study of the conversion of [Ru(bpy)_2_(pbn)]^2+^ into [Ru(bpy)_2_(pbnHH)]^2+^ (Polyansky et al., 2008). Complex **1HH**, which is the photoreduction product of **1** in the presence of a sacrificial reagent, is diamagnetic, which is consistent with the results of the ESR measurements in the case of photoirradiation for 900 s.

**Scheme 1:**
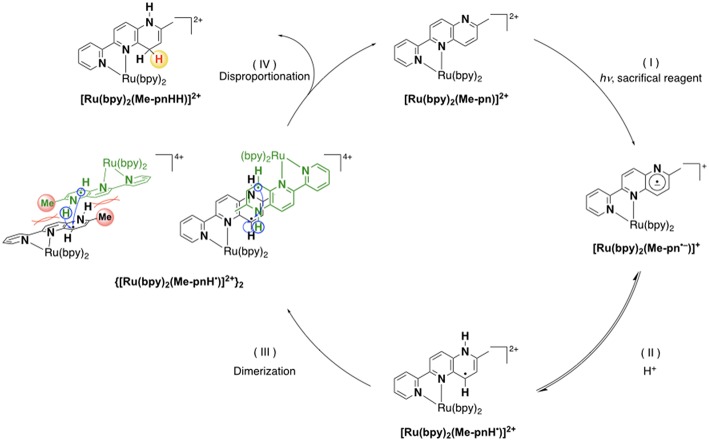
A possible reaction mechanism for the photo-driven NAD^+^/NADH-type hydrogenation reaction from **1** to **1HH**.

Based on the various experimental results described above and the previously reported mechanism for the photo-driven NAD^+^/NADH-type hydrogenation reaction using [Ru(bpy)_2_(pbn)]^2+^ (Polyansky et al., 2008), a photo-driven hydrogenation reaction pathway of **1** into **1HH** in the presence of a sacrificial reagent under photoirradiation can be proposed, as shown in Scheme 1. First, the NAD^+^-type complex **1** is photochemically reduced to the Me-pn radical anion species [Ru(bpy)_2_(Me-pn^•−^)]^+^ (**1**^**•−**^) by the reductive quenching of the photoexcited 1^*^ by a sacrificial donor such as TEOA or TEA (step I). Secondly, the protonation of the non-coordinating N atom of the Me-pn^•−^ in **1**^**•−**^ produces [Ru(bpy)_2_(Me-pnH^•^)]^2+^ (**1**^**•−**^**H**) radical species (step II). Then, each **1**^**•−**^**H** dimerizes via the π-π stacking of the neutral Me-pnH^•^ moieties to afford {[Ru(bpy)_2_(Me-pnH^•^)]^2+^}_2_ ((**1**^**•−**^**H)**_**2**_) (step III). Finally, intramolecular proton-coupled electron transfer from one Me-pnH^•^ in (**1**^**•−**^**H)**_**2**_ to another leads to disproportionation, thus generating the **1HH** NADH-type complex and regenerating the **1** NAD^+^-type complex. At present, we have yet to clarify the reason why there is a significant difference in the photo-driven NAD^+^/NADH-type hydrogenation reaction between **1** and the previously reported [Ru(bpy)_2_(pbn)]^2+^ system. However, the steric hindrance of the methyl groups in Me-pn complex may be the reason why we can see the radical intermediate species such as **1**^**•−**^**H** in the ESR measurements more easily than for the previously reported [Ru(bpy)_2_(pbn)]^2+^.

## Conclusions

In conclusion, the present study has demonstrated the synthesis, characterization, and photoinduced NAD^+^/NADH conversion properties of a new ruthenium(II) complex, [Ru(bpy)_2_(Me-pn)](PF_6_)_2_ (**1**), as well as the isolated NADH-type complex [Ru(bpy)_2_(Me-pnHH)](PF_6_)_2_ (**1HH**) under photochemical reduction conditions. The findings of the present study suggest a new type of photo-driven NAD^+^/NADH-type hydrogenation reaction that makes possible the development of photoinduced CO_2_ reduction reactions utilizing the NAD^+^/NADH redox function. These results provide valuable information for the further development of functional transition-metal NAD^+^/NADH-type complexes and shed new light on the applications of the NADH-type complexes for future catalysts and energy storage materials in the area of artificial photosynthesis. Further investigations concerning photochemical reduction of CO_2_ and other substrates by using the NAD^+^/NADH-functionalized complexes are now in progress.

## Data Availability

The datasets generated for this study can be found in Cambridge Crystallographic Data Center, CCDC 1894384.

## Author Contributions

HO directed the project, conceived and designed the experiments, supervised the progress of this work, and wrote the manuscript. TS carried out the syntheses of ligands and complexes and performed most of the experiments. KT contributed X-ray structural analyses. All authors listed have discussed the results, drawn conclusions, and given the approval to the final version of the manuscript.

### Conflict of Interest Statement

The authors declare that the research was conducted in the absence of any commercial or financial relationships that could be construed as a potential conflict of interest.
