# Supplementary material for: A Novel Photo-Driven Hydrogenation Reaction of an NAD+-Type Complex Toward Artificial Photosynthesis
Source: Front Chem. 2019 Aug 20;7:580. doi: 10.3389/fchem.2019.00580 (PMC6710353; doi:10.3389/fchem.2019.00580)
Supplement: Supplementary file 1 [file Data_Sheet_1.PDF]

*Supplementary Material*

# A Novel Photo-Driven Hydrogenation Reaction of an $NAD^+$-Type Complex toward Artificial Photosynthesis

**Hideki Ohtsu*, Tsubasa Saito, and Kiyoshi Tsuge**

*** Correspondence:** Dr. Hideki Ohtsu: ohtsu@sci.u-toyama.ac.jp

*Graduate School of Science and Engineering, University of Toyama, 3190 Gofuku, Toyama 930-8555, Japan*

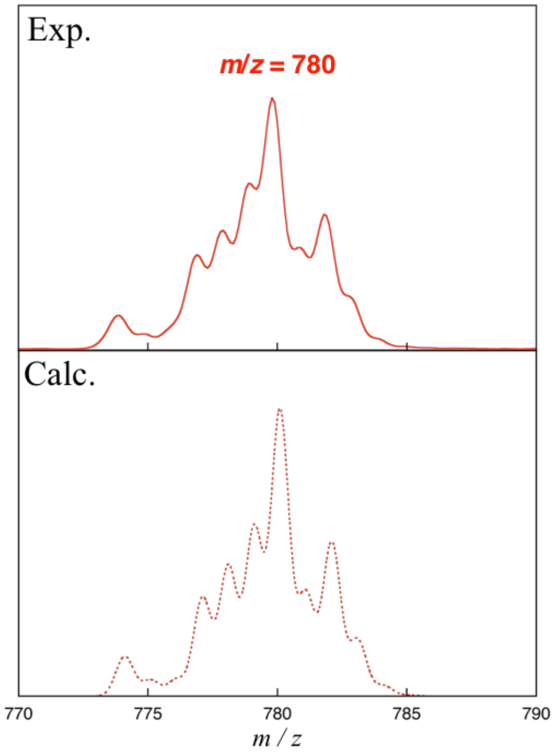


**Figure S1.** Observed (top) and calculated (bottom) ESI mass spectra of **1** in $CH_3CN$ at 298 K.

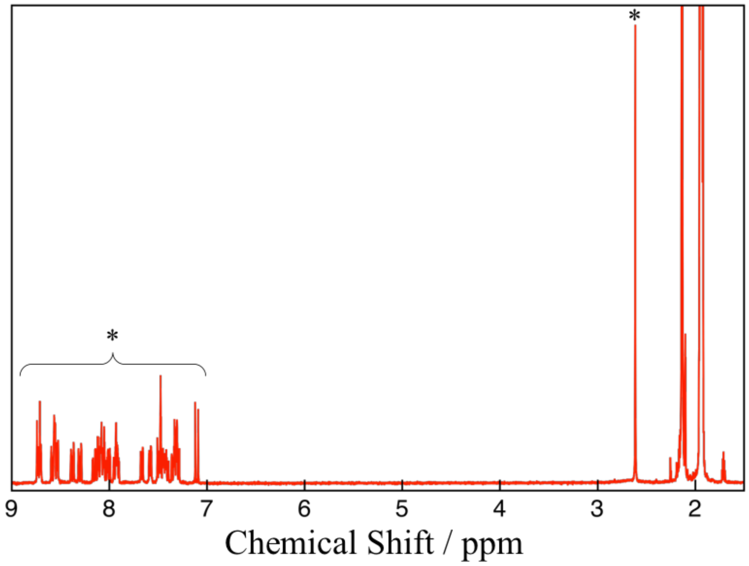


**Figure S2.** $^{1}$H-NMR spectrum of **1** in $CD_3CN$ at 298 K. Peaks corresponding to **1** are denoted by asterisks.

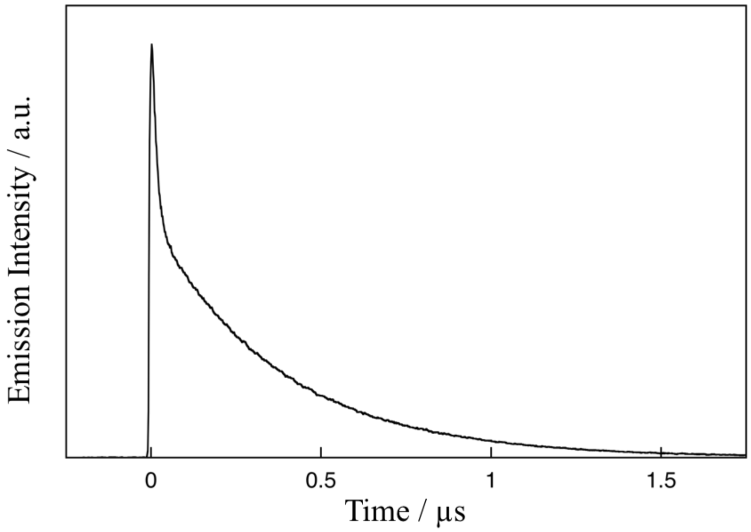


**Figure S3.** Emission decay curve of **1** in $CH_3CN$ measured at 685 nm at 298 K ($\lambda_{ex}$ = 337 nm).

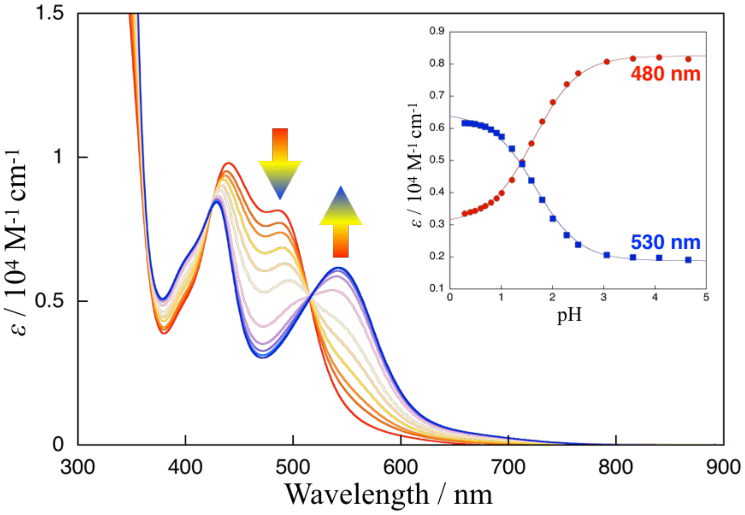


**Figure S4.** The pH-dependent absorption spectra of **1** in $H_2O$ from pH 4.65 to pH 0.29 at 298 K. Inset: the pH titration curves obtained by plotting the absorbances at 480 nm (red circle) and 530 nm (blue square).

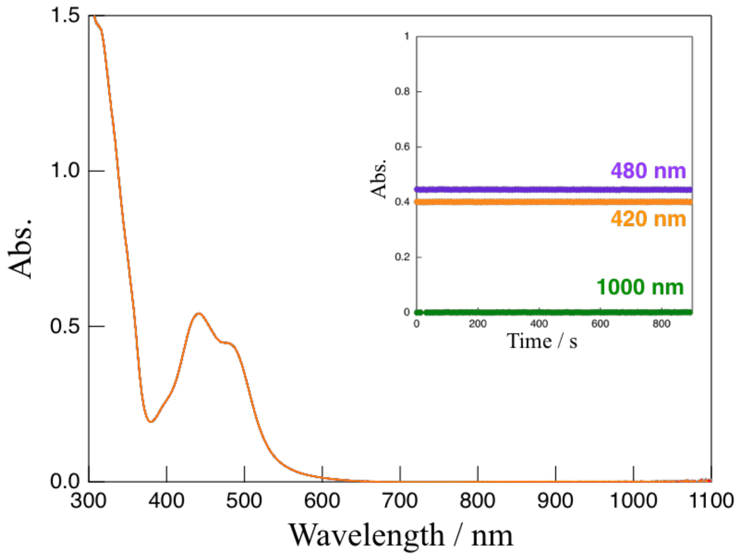


**Figure S5.** Absorption spectral changes observed upon the irradiation with visible light ($\lambda > 420$ nm) to a $CH_3CN$ solution of **1** at 293 K from 0 to 900 s. Inset: time course of the absorption changes at 420, 480, and 1000 nm.

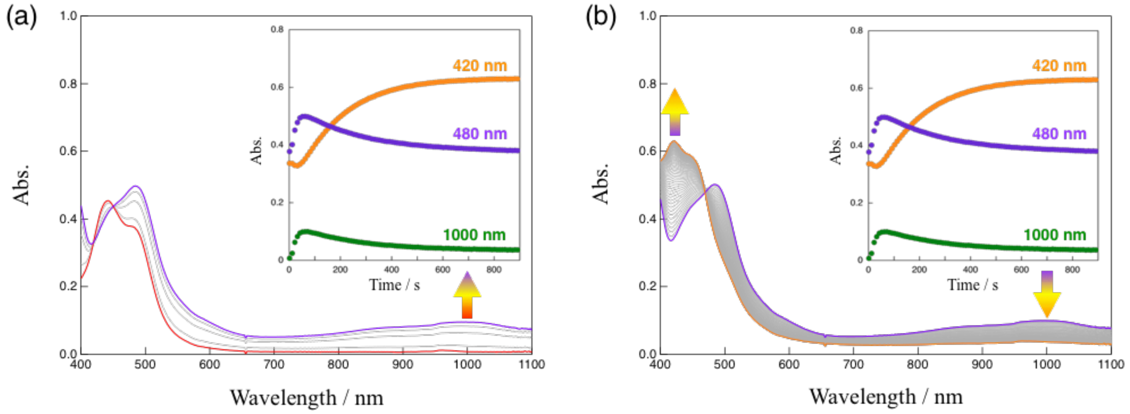


**Figure S6.** Absorption spectral changes observed upon the irradiation with visible light ($\lambda > 420$ nm) to a solution ($CH_3CN$:TEA:$H_2O$ = 98:1:1 v/v) of **1** at 293 K from 0 to 50 s (a) and from 50 to 900 s (b), respectively. Inset: time course of the absorption changes at 420, 480, and 1000 nm.

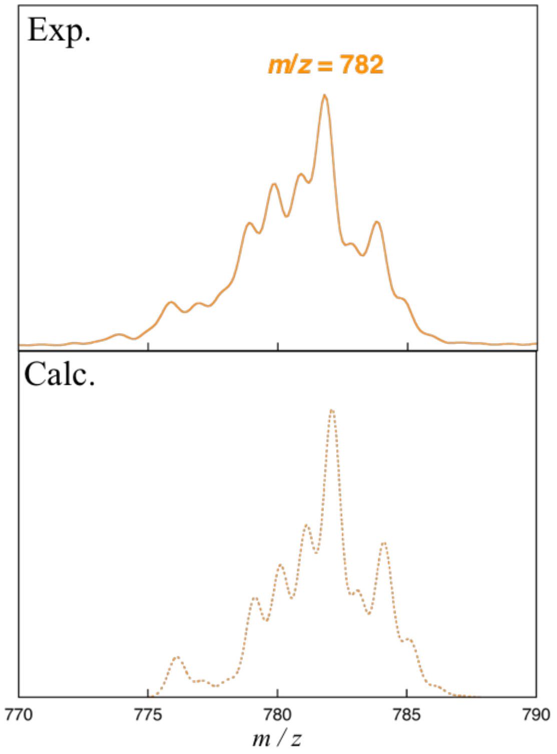


**Figure S7.** ESI mass spectrum of **1** in $CH_3CN$:TEA:$H_2O$ = 98:1:1 v/v solution after photoirradiation ($\lambda$ > 420 nm) for 900 s at 293 K (top). Calculated natural isotope abundance pattern for **1HH** (bottom).

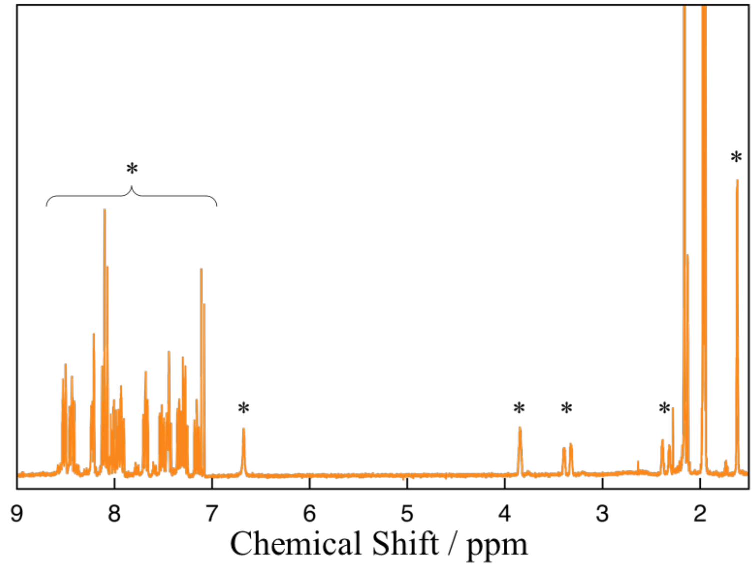


**Figure S8.** $^1$H-NMR spectrum of **1HH** in $CD_3CN$ at 298 K. Peaks corresponding to **1HH** are denoted by asterisks.
